# 2408 Genital microbiomes of women with recurrent bacterial vaginosis and their regular male sexual partner

**DOI:** 10.1017/cts.2018.78

**Published:** 2018-11-21

**Authors:** Christina A. Muzny, William J. Van Der Pol, Elliot J. Lefkowitz, Arindam Ghosh, Mei Li, David Redden, Xiangqin Cui, Jane Schwebke

**Affiliations:** University of Alabama at Birmingham

## Abstract

OBJECTIVES/SPECIFIC AIMS: Epidemiologic data suggest that BV is sexually transmitted with male partners colonized or infected with the responsible organism(s). The objective of this study was to compare the genital microbiota of women with recurrent BV and their regular male sexual partner using 16S rRNA gene sequencing and quantitative PCR targeting BV-candidate bacteria (*Gardnerella vaginalis*, *Atopobium vaginae*, BVAB1-3, Sneathia, Leptotrichia, and Megasphaera type I). METHODS/STUDY POPULATION: Women with recurrent BV (≥3 prior episodes, including a current episode) and their regular male partner participating in a BV treatment trial and providing genital specimens (women: vaginal; men: urethral, coronal sulcus, urine) at enrollment were included. Male specimens for each participant were pooled. 250 bp 16S rRNA V4 region PCR amplicons were sequenced and analyzed using the QIIME pipeline. Taxonomy was assigned using the RDP Classifier against a modified Greengenes database with additional vaginal taxonomies added. An average relative abundance cutoff of 0.5% was used for analysis. qPCR was also performed for specific BV-candidate bacteria. Spearman correlation coefficients were used to investigate associations between all genital bacteria in addition to BV-candidate bacteria between partnerships. To determine positive associations between partnerships, the Wilcoxon signed-rank test was used. RESULTS/ANTICIPATED RESULTS: In total, 45 partnerships were included. Mean partnership age was 31.3 (SD=7.9), 91.1% partnerships were African-American. The majority of partnerships (70.0%) reported condomless sex during the past 3 months. Regarding 16S data, 37 genital bacteria had an average relative abundance of ≥0.5%. The average Spearman correlation across all 45 partnerships was 0.28 (SD=0.27) (median=0.27, minimum=−0.21, maximum=0.84). Overall, a positive association of all genital bacteria existed across the partnerships (*p*<0.0001). However, regarding specific BV-candidate bacteria, Spearman correlation tests for *G. vaginalis*, *A. vaginae*, *Prevotella bivia*, Megasphaera type I, BVAB1, and BVAB2 were nonsignificant. In contrast, *Sneathia* spp. were positively correlated between partnerships (*r*=0.37, *p*=0.01). With regards to qPCR results, RNA Cq analyses provided significant evidence for a linear association between male and females for only *A. vaginae* (*r*=0.52, *p*=0.006). DISCUSSION/SIGNIFICANCE OF IMPACT: In monogamous heterosexual couples in which the female has BV, the vaginal microbiota of women and the penile/urine microbiota of men were significantly correlated, particularly with regards to *Sneathia* spp. and *A. vaginae*, supporting the hypothesis that BV-associated bacteria are exchanged during sex.

